# Kashmiri refugees at the verge of hepatitis B and C epidemic in the State of Azad Jammu and Kashmir, Pakistan

**DOI:** 10.11606/s1518-8787.2022056003479

**Published:** 2022-04-27

**Authors:** Syed Ayaz Kazmi, Abdul Rauf, Farheen Shafique, Noreen Asim, Nuzhat shafi, Mahreen Ul Hassan

**Affiliations:** I University of Azad Jammu and Kashmir Department of Zoology Muzaffarabad Pakistan University of Azad Jammu and Kashmir. Department of Zoology. Muzaffarabad, Pakistan; II Institute of Biotechnology and Genetic Engineering Division of Genomics and Bioinformatics The University of Agriculture Peshawar Pakistan Institute of Biotechnology and Genetic Engineering. Division of Genomics and Bioinformatics. The University of Agriculture, Peshawar, Pakistan; III Shaheed Benazir Bhutto Women University Department of Microbiology Peshawar Pakistan Shaheed Benazir Bhutto Women University. Department of Microbiology. Peshawar, Pakistan

**Keywords:** Refugees, Hepatitis B, Hepatitis C, Risk Factors, Seroepidemiologic Studies

## Abstract

**OBJECTIVE:**

To determine the seroprevalence of hepatitis B and C among immigrants residing refugee camps in Muzaffarabad, Azad Kashmir, Pakistan, and to identify possible risk factors for hepatitis B virus (HBV) and hepatitis C virus (HCV) transmission.

**METHODS:**

Around 1,225 individuals inhabiting Muzaffarabad refugee camps, participated in the study. A qualitative Immuno-Chromatographic Technique was used for initial screening and PCR test was used for detection of HBV and HCV in participants. The major risk factors for HBV and HCV transmission were assessed using a questionnaire approach.

**RESULTS:**

Around 86 (7.0%) individuals were observed for hepatitis B surface antigen (HBsAg) presence, and 215 (17.5%) individuals were found positive for Anti-HCV. Only 32 (2.6%) individuals were confirmed for HBV DNA and 126 (10.3%) individuals were positive for HCV RNA after PCR. Demographically, both HBsAg and Anti-HCV were found more prevalent in female (4.4% HBsAg and 10.8% Anti-HCV) population as compared to male (2.6% HBsAg and 6.7% Anti-HCV) population. Surprisingly, the HBsAg (23.5%) and Anti-HCV (41.1%) appeared to be more frequent in the age group 62–75 years. Previous history of hepatitis in the family (p < 0.0001), blood transfusion (p = 0.0197) dental treatment (p < 0.0001) and tattooing or piercing on any part of the body (p = 0.0028) were assessed as significant risk factors in HBV and HCV transmission.

**CONCLUSIONS:**

Presence of 7.0% HBsAg and 17.5% Anti-HCV in a small fragment of the migrant population cannot be overlooked. Lack of awareness among people and negligence of health department could escalate the situation.

## INTRODUCTION

In comparison to other regions of the globe, Pakistan has limited resources and scant surveillance to determine the true extent of viral hepatitis among refugees. Hepatitis B and C are very complex viral infections which could easily spread from infected individual to other through contaminated blood or body fluids^1^. The viruses responsible for hepatitis B and C infections are considered as not only the major public health concern but also an economic burden with millions of people having chronic infection worldwide^2^. The commonly found liver cancer is hepatocellular carcinoma (HCC) and it is mostly caused by viral hepatitis B or C infection^3^. Many studies have shown that carriers of hepatitis B surface antigen (HBsAg) have higher risk of HCC compared to HBsAg negative cases^4^. Several other studies showed that hepatitis B and C infections may increase the risk of lymphoid malignancies^5^, pancreatic cancer^6^, liver fibrosis^7^ and chronic kidney disease^8^. Moreover, studies have shown that hepatitis C virus (HCV) is present not only in hepatocytes, but also in patients’ plasma and serum. Although hepatocytes are the key target for HCV, viral RNA may also be found in extrahepatic compartments^9^.

The hepatitis B virus (HBV) is estimated to be one of the most common diseases worldwide, and about 150 million people suffer from chronic hepatitis B infection. Its geographical distribution varies greatly; high prevalence areas include Eastern Europe, Southeast Asia, China and sub-Saharan Africa. While HCV infection is cosmopolitan, affecting approximately 3% of the world population, the areas of highest prevalence include countries of the Far East, the Mediterranean basin and certain areas of Africa and Europe. Millions of people die due to these infections, with a frequency of one in twelve individuals around the globe^10^.

Refugee communities are more vulnerable to HBV and HCV infections due to a lack of access to health care facilities and an inability to obtain information on the spread and prevention of these diseases from various sources^11^.

In the USA, among immigrants, 16.7% of the tested population (89/534) was found with HBsAg prevalence, including 41 males and 48 females^12^.

In the past few years, refugees from many countries have arrived in Greece^13^, with a high incidence of viral hepatitis, Aids, and tuberculosis^13^; high prevalence of HBV but low incidence of HCV was observed in the refugees of Athens^14^. Hepatitis B has been reported to be prevalent among Afghan refugees in Pakistan’s Baluchistan camps. Many risk factors are associated with the infection such as lack of knowledge, contaminated blood transfusion, and vertical transmission and in addition, the reuse of syringes will continuously rapidly increase the spread of this infection until specific control measurements are taken^15^.

This study aimed to determine the frequency of hepatitis B and C infections among refugees living in various refugee camps in Muzaffarabad, Azad Kashmir, Pakistan, and analyse the risk factors that contribute to the transmission of these viruses.

## METHODS

### Scheme of Study

A “random cross-sectional study” was designed and conducted among a vulnerable population of refugees who migrated from the Indian occupied Kashmir in 1989 to Azad Kashmir, Pakistan. The study was carried out between 15 January 2014 to 20 April 2019, and was reported in compliance with the STROBE guidelines, also described by Von et al.^16^ (2014).

### Population, Sample Size and Eligibility Criteria

In this random cross-sectional study, the sample size was calculated using Raosoft software. About 19,000 refugees were inhabiting in three different refugee camps (Manakpayyan camp, Ambor camp, and Nisar camp) around Muzaffarabad, Azad Jammu and Kashmir administered by Pakistan. The response distribution was set at 50%, while the confidence interval was set at 95% and the error margin at 5%. The minimum recommended sample size after analysis was 377. Screening camps were organized and 1,225 participants (including 585 males and 640 females) with an age ranging between 20 to 75 years participated in the study. Individuals with other liver infections were excluded, as well as individuals who migrated before 1989 and the participants who refused to give any details.

### Data Collection and Management

During population screening, all the participants were interviewed via the questionnaire process. Before sampling, each individual participating provided an informed consent form, which was focused on the compilation of demographic data and questions related to possible risk factors. The participants filled out a pre-tested questionnaire at their home for their convenience.

### Sampling and Initial Screening

A gel tube (Biotube Gel Clot Activator) was used to collect around 5mL of blood from each individual. After centrifugation, cells were discarded, and the labelled tubes containing serum were stored at -20 °C. For rapid detection of HBV and HCV Immuno-Chromatographic Technique (ICT) kits (SD BIOLINE HBsAg and Anti-HCV kits) were used, which detect the presence of hepatitis B surface antigen (HBsAg) and hepatitis C virus antibody (anti-HCV) in serum.

### HBV DNA Isolation and Gene Amplification

The viral DNA was extracted from the blood samples of HBsAg-positive individuals via remoulded DNAzol method optimised by Rauf et al.^17^ (2013). For the confirmation of hepatitis B and C viruses in the blood, PCR reactions were performed.

### HCV RNA Extraction, Amplification, and Genotyping

To detect the hepatitis C Virus, viral RNA is extracted from serum of HCV-ICT positive individuals using TRIzol RNA Isolation protocol described by Chomczynski et al.^18^ (1995). Followed by Real Time PCR, which was performed using Favor-PrepTM Viral purification kit (Favorgen®, Biotech Corp, USA) and Verso 1-Step RT-PCR Hot Start Kit (Thermo Scientific®, USA). The PCR amplifies 5` UTR of HCV genome by using a set of specific primers previously used by Casanova et al.^19^ (2014). The quantification range of the assay was 100IU/ml to 350,000,000IU/ml. All HCV genotypes i.e., 1a, 1b, 2a, 2b, 2c, 3a, 3b, 4, 5a, and 6. were amplified with equal efficacy.

### Statistical Analysis

The data collected was statistically analysed for frequency distribution and the relationship between risk factors and the spread of HBV and HCV infections. All the analysis was carried out with Microsoft Excel 2016, SPSS 26.0 and GraphPad Prism 9.0.

## RESULTS

**Figure 1:**
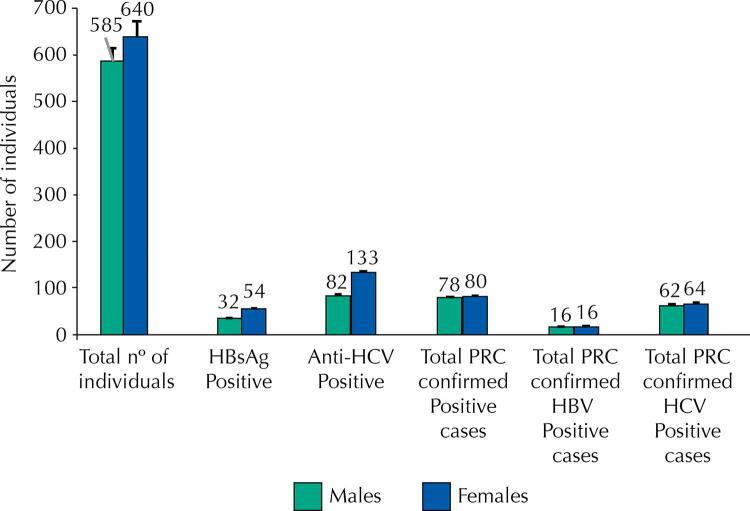
Overall prevalence of hepatitis B and C in refugees.

About 1,225 individuals were screened for HBV and HCV in this study, including 585 males and 640 females. Out of these 1,225 participants, 86 (7.0%) individuals tested positive for HBsAg and 215 (17.5%) individuals tested positive for Anti-HCV through qualitative ICT. These positive cases were further subjected to RT-PCR for confirmation of active HBV and HCV infections. The RT- PCR confirmed 32 (16 males and 16 females) individuals for HBV and 126 (62 males and 64 females) individuals for HCV infection, as Figure 1 shows.

7.0% refugees were found HBsAg positive and 17.5% refugees were found anti-HCV positive. Both, HBsAg and anti-HCV were found more prevalent in female (4.4% HBsAg and 10.8% anti-HCV) population as compared to male (2.6% HBsAg and 6.7% anti-HCV) population.

In this study, Table 1 shows the participants were split into four age groups. Out of 86 HBsAg positive individuals, 36 (9.7%) individuals were found in the age group 20–33 years (n = 372), 24 (4.8 %) were found within 34–47 years (n = 500), 22 (6.5 %) were found within 48–61 years (n = 336) and 4 (23.5 %) were found within 62–75 years age group (n = 17). Similarly, out of 215 anti-HCV positive individuals, 96 (25.8 %) were observed in the group having participants aged 20 to 33 years, 78 (15.6 %) were found within 34–47 years, 34 (10.1 %) were found in 48–61 years, and 7 (41.1 %) were found within 62–75 years, age group (n = 17). Surprisingly, the HBsAg (23.5%) and Anti-HCV (41.1%) were appeared to be more frequent in the age group 62–75 years.

**Table 1:** Prevalence of hepatitis B and C among refugees with respect to age groups.

Age	Number of Individuals (%)	Mean Age with 95%CI	HBsAg Positive (%) (n = 1,225)	HBV Positive confirmed through PCR^a^ (%) (n = 86)	Anti-HCV Positive (%) (n = 1,225)	HCV Positive confirmed through PCR^b^ (%) (n = 215)
20–33	372 (30.4 %)	27.84 ± 0.51	36 (9.7 %)	16 (4.3 %)	96 (25.8 %)	62 (16.6 %)
34–47	500 (40.8 %)	41.15 ± 0.49	24 (4.8 %)	12 (2.4 %)	78 (15.6 %)	44 (8.8 %)
48–61	336 (27.4 %)	52.11 ± 0.42	22 (6.54 %)	4 (1.2 %)	34 (10.1 %)	16 (4.76 %)
62–75	17 (1.4 %)	68.33 ± 4.49	4 (23.5 %)	0 (0.0 %)	7 (41.2 %)	4 (23.5 %)
Total Individuals	1, 225	40.38 ± 0.82	86 (7.0 %)	32 (2.6 %)	215 (17.5 %)	126 (10.3 %)

^a^ HBV confirmed positive after PCR Test of HBsAg positive individuals.

^b^ HCV confirmed positive after PCR Test of Anti-HCV positive individuals.

Among 1,225 total participants, 600 individuals were selected from Manakpayyan camp, 320 from Ambor camp and 305 individuals from Nisar camp (Table 2). In the Manakpayyan camp, 45 (7.5%) individuals were found HBsAg positive, out of which 08 (1.3%) individuals were found positive for HBV through PCR. While, 114 (19%) individuals were found anti-HCV positive through ICT test, out of which 92 (15.3%) individuals were found positive for HCV through PCR.

**Table 2:** Prevalence of hepatitis B and C among refugees of different camps.

Clusters	Number of Individuals	HBsAg Positive	HBV Positive	Anti-HCV Positive	HCV Positive
Manakpayyan Camp	600	45 (7.5%)	08 (1.3%)	114 (19%)	92 (15.3%)
Ambor Camp	320	26 (8.1%)	20 (6.2%)	56 (17.5%)	28 (8.7%)
Nisar Camp	305	15 (4.9%)	04 (1.3%)	45 (14.7%)	06 (1.9%)
Total	1225	86 (7.0%)	32 (2.6%)	215 (17.5%)	126 (10.3%)

Similarly, in the Ambor camp, 26 (8.1%) individuals tested HBsAg positive through ICT test, out of which 20 (6.2%) individuals tested positive for HBV through PCR. While, 56 (17.5%) individuals tested Anti-HCV positive through ICT test, out of which 28(8.7%) individuals were confirmed for HCV via PCR.

In the case of Nisar camp, 15 (4.9%) individuals tested HBsAg positive, out of which 04 (1.3%) individuals tested positive for HBV through PCR. Similarly, 45 (14.7%) individuals tested anti-HCV positive, out of which 06 (1.9%) individuals tested positive for HCV by PCR. The study indicates a high prevalence of HCV as compared to HBV in all the refugee camps.

**Figure 2:**
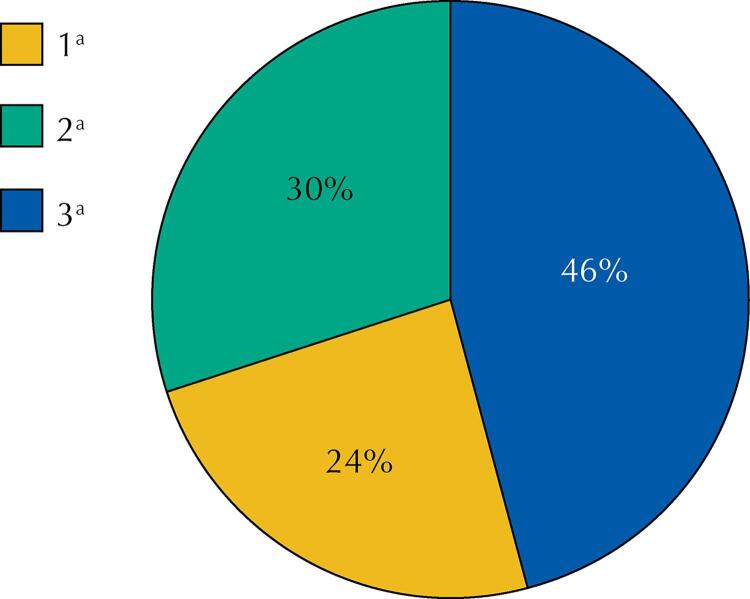
Prevalence of hepatitis C (1a, 2a and 3a) genotypes among HCV positive refugees.

In genotype analysis of 126 HCV positive participants, genotype 1a was found in 30 (24%) individuals, genotype 2a was found in 38 (30%) individuals, and genotype 3a was found in 58 (46%) individuals, as Figure 2 shows.

To assess the significance of each risk factor responsible for the spread of hepatitis B and C in Kashmiri refugees, Chi-square test was conducted with 95% confidence interval at p-value 0.05 (Table 03). For confirmation, the results were further analyzed through Fisher’s exact test by using GraphPad Prism (version 9.0). Previous history of hepatitis in the family (p < 0.0001), blood transfusion (p = 0.0197) dental treatment (p < 0.0001) and tattooing or piercing on any part of the body (p = 0.0028) were found significant through Chi-square test. While, admit to hospital for any reason (p = 0.2732) was found not significant. However, on the data examined for injection history, HBV immunisation, and transplantation, the Chi-square test was found to be inapplicable.

**Table 3:** Summary of risk factors associated with spread of hepatitis B and C among refugees.

Factors	Responses	Overall responses (n = 1,225)	HbsAg & Anti-HCV Positive individuals (n = 301)	HbsAg & Anti-HCV Negative individuals (n = 924)	Chi-square value (at p = 0.05)
Previous hepatitis history or jaundice in family	Yes	60 (4.8 %)	34	26	p < 0.0001
	No	1,165 (95.1 %)	267	898	
HBV vaccination	Yes	0	0	0	N/A
	No	1,225 (100 %)	301 (100 %)	924 (100 %)	
Transfusion of blood	Yes	32 (2.6 %)	14	18	p = 0.0197
	No	1,193 (97.4 %)	287	906	
Visit to dental clinic	Yes	156 (13 %)	86	70	p < 0.0001
	No	1,069 (87.2 %)	215	854	
Tattooing or piercing on any part of the body	Yes	740 (60.4 %)	204	536	p = 0.0028
	No	485 (40.0 %)	97	388	
Injections history	Yes	1,225 (100 %)	301	924	N/A
	No	0	0	0	
Admit to hospital for any reason	Yes	58 (4.7 %)	18	40	p = 0.2732
	No	1,167 (95.2 %)	283	884	
Transplantation	Yes	0	0	0	N/A
	No	1,225 (100 %)	301	924	

N/A: not applicable.

## DISCUSSION

Pakistan is one of the few countries in the world who accepts refugees open-heartedly. But this generosity always comes with a price in terms of over-population, lack of resources and disease. The study was designed to determine the frequency of viral hepatitis B and C, as well as relevant risk factors, among Kashmiri refugees. Quddus et al.^15^ (2006) conducted a study on Afghan refugees living in Baluchistan (Pakistan), focused on HBsAg prevalence only. The frequency of HBsAg among the population of Afghan refugees residing in different camps in Baluchistan was found to be 8.3 %. In comparison, this study showed low (7.0 %) prevalence of HBsAg in among the refugees inhabiting in refugee camps around Muzaffarabad. However, we also assessed the prevalence of anti-HCV and it was found higher (17.5 %) among Kashmiri refugees.

Roussos et al.^14^ (2003) were the first to report the prevalence of HBsAg (15.4%) and anti-HCV (2.3%) among refugees living in Athens. While in Greece, several reports have depicted the role of HCV in liver cirrhosis and hepatocellular cancer^20,21^. In this study, we found 7.0% prevalence of HBsAg among Kashmiri refugees which is 2.2 times lower than 15.4% prevalence of HBsAg in Athen. However, as compared to 17.5% prevalence of anti-HCV among Kashmir refugees, the 2.3 % seroprevalence of anti-HCV among refugees residing in Athens, is quite low.

Individuals aged 30 years or above were more likely to have HCV antibodies (5.6 %) as compared to the individuals aged 18 years (0.8 %)^22^. While, this study revealed a higher prevalence of HCV antibodies (25.8 %) in the age-group 20–33 years. However, the individuals having age below 20 years were excluded from the present study. But, Quddus et al.^15^ (2006) reported 10.9% prevalence of HBsAg among the afghan refugees of 41–50 years age group and it was higher among all other age groups. Though, in our study we found 4.8 % prevalence of HBsAg in 34–47 years age group and 6.5 % among individuals of 48–61 years age group.

The detection rate of HBsAg and anti-HCV in female refugees in Gambella, Ethiopia was 6.8% and 1.4 percent, respectively, while it was 9.6% and 4.8% among males respectively^23^. Whereas, in this study, both HBsAg and anti-HCV were found more prevalent in female (4.4% HBsAg and 10.8% anti-HCV) population as compared to male (2.6% HBsAg and 6.7% anti-HCV) population. The difference between both studies is might be due to very low male population size as compared to high female population size of the study of Ayele et al.^23^ (2020).

The risk of HBV infection rose among Afghan immigrants in Baluchistan, Pakistan, after reusing syringes several times during the preceding year^15^. Proposed risk factors like marital status, the interchange of sharp materials among persons, and multiple sexual partners, were not identified to be risk factors for HBV and HCV infections among Gambella refugees, Ethiopia^23^. History of dentist visits, transfusion, surgery, and unprotected sexual contact were the major risk factors in Peshawar, Pakistan, which contributed to the rise in incidence of hepatitis B and C^24^. However, in this study, previous history of hepatitis in the family (p < 0.0001), blood transfusion (p = 0.0197), dental treatment (p < 0.0001) and tattooing or piercing on any part of the body (p = 0.0028) were found significant through Chi-square test. While, hospitalization for any reason (p = 0.2732) was found non-significant. Whereas, the Chi-square test was not found applicable on the data assessed for the history of injection, HBV vaccination and transplantation.

In a review study done in Pakistan, Haqqi et al.^25^ (2019) found that HCV genotype 3a (58.16 %) was the most common, followed by genotypes 3b (9.05 %), 2a (6.70 %), 1a (6.22 %), and 1b (2.39 %). This study among refugees in Muzaffarabad also declares that among 126 HCV positive individuals, genotype 3a (46%) was more frequent genotype of HCV followed by genotypes 2a (30%) and 1a (24%).

We concluded that 7.0% prevalence of HBsAg and 17.5% prevalence of anti-HCV in a small fragment of the migrant population cannot be neglected. This research revealed that the current prevalence of viral hepatitis in the refugee camps might further be increased if immediate action is not taken. The higher prevalence of viral hepatitis in female population also indicates the maternal transfer of these viruses among their children. So, further studies should be conducted to evaluate the exact prevalence of hepatitis B and C and associated risk factors among the whole refugee population.
